# Spatial prioritisation for conserving ecosystem services: comparing hotspots with heuristic optimisation

**DOI:** 10.1007/s10980-015-0258-5

**Published:** 2015-09-02

**Authors:** Matthias Schröter, Roy P. Remme

**Affiliations:** Environmental Systems Analysis Group, Wageningen University, P.O. Box 47, 6700 AA Wageningen, The Netherlands; Department of Ecosystem Services, UFZ – Helmholtz Centre for Environmental Research, Permoserstr. 15, 04318 Leipzig, Germany; German Centre for Integrative Biodiversity Research (iDiv) Halle-Jena-Leipzig, Deutscher Platz 5e, 04103 Leipzig, Germany

**Keywords:** Hot spot, Mapping, Modelling, Overlap

## Abstract

**Context:**

The variation in spatial distribution between ecosystem services can be high. Hence, there is a need to spatially identify important sites for conservation planning. The term ‘ecosystem service hotspot’ has often been used for this purpose, but definitions of this term are ambiguous.

**Objectives:**

We review and classify methods to spatially delineate hotspots. We test how spatial configuration of hotspots for a set of ecosystem services differs depending on the applied method. We compare the outcomes to a heuristic site prioritisation approach (Marxan).

**Methods:**

The four tested hotspot methods are top richest cells, spatial clustering, intensity, and richness. In a conservation scenario we set a target of conserving 10 % of the quantity of five regulating and cultural services for the forest area of Telemark county, Norway.

**Results:**

Spatial configuration of selected areas as retrieved by the four hotspots and Marxan differed considerably. Pairwise comparisons were at the lower end of the scale of the Kappa statistic (0.11–0.27). The outcomes also differed considerably in mean target achievement, cost-effectiveness in terms of land-area needed per unit target achievement and compactness in terms of edge-to-area ratio.

**Conclusions:**

An ecosystem service hotspot can refer to either areas containing high values of one service or areas with multiple services. Differences in spatial configuration among hotspot methods can lead to uncertainties for decision-making. This also has consequences for analysing the spatial co-occurrence of hotspots of multiple services and of services and biodiversity.

**Electronic supplementary material:**

The online version of this article (doi:10.1007/s10980-015-0258-5) contains supplementary material, which is available to authorized users.

## Introduction

The concept of ecosystem services (ESs) encompasses multiple contributions of ecosystems to human well-being (Haines-Young and Potschin 2010). It is increasingly being used to analyse the human-nature relationship and to inform policymaking (Carpenter et al. 2009; Larigauderie et al. 2012). An important approach to assess biophysical quantities of multiple ES has been spatial modelling and mapping (Maes et al. 2012a; Martínez-Harms and Balvanera 2012; Nemec and Raudsepp-Hearne 2013; European Commission 2014). These spatial ES assessments could be used for systematic conservation planning to ensure the long-term capacity of ecosystems to provide services (Egoh et al. 2007). Considering ESs in conservation planning is, however, a fairly new practice, which still needs to be operationalized (Chan et al. 2011; Luck et al. 2012b; Cimon-Morin et al. 2013). The advantage of this approach is that it seeks for a way to combine biodiversity conservation with the provision of ESs that originate from natural or semi-natural ecosystems.

Spatial distribution and abundance of ESs across the landscape is spatially heterogeneous and differs between ESs (Egoh et al. 2008; Raudsepp-Hearne et al. 2010; Bai et al. 2011). Different degrees of spatial overlap between ES increase the complexity of conservation planning. Hence, there is a need to identify important sites for conservation of multiple ES (Luck et al. 2012b), for instance in order to select sites for new protected areas. The term ‘ES hotspot’ is increasingly used for the purpose of informing spatial prioritisation of ES (Cimon-Morin et al. 2013). For instance, the number of studies containing the terms “ecosystem service*” and “hotspot*” in title, abstract and keywords increased from 9 in 2006 to 39 in 2013 (Scopus search, 30 October 2014). Despite this growing use of the term, ES hotspot is not clearly defined in the literature yet. While the earlier established notion of a biodiversity hotspot has been defined as an area of both high biodiversity and high level of threat (Myers 1988, 1990; Mittermeier et al. 1998; Myers et al. 2000), the use of the term ES hotspot in the literature differs from that notion. ES hotspot often refers to an area where high amounts of one particular service are present (Cimon-Morin et al. 2013), but other studies have defined hotspots as areas where multiple ESs overlap (e.g., Gos and Lavorel 2012). Spatial configuration of selected sites might differ depending on the hotspot method applied. As this could lead to inconclusive recommendations to decision makers more clarity is needed on the variety of different existing approaches and their potential advantages and shortcomings. Different hotspot methods might serve different policy purposes, which need to be clarified and discussed. Furthermore, it is unclear to what extent site prioritisation based on hotspots complies with principles of systematic conservation planning (Margules and Pressey 2000; Possingham et al. 2006), such as comprehensiveness, cost-effectiveness and compactness of the spatial arrangements of selected sites. The conservation software Marxan has been developed to select sites for conservation according to these principles and is based on a heuristic optimisation algorithm (Ball et al. 2009). Marxan prioritises sites to protect proportions of the total amount of conservation feature in an area, e.g. a species or an ES. These relative targets can refer to both presence data (e.g., a certain proportion of the habitat area of a service-providing species) and metric data (e.g., a proportion of total amount of carbon stored in an area). Marxan has recently been applied to integrate ESs in different conservation problems (Chan et al. 2006, 2011; Egoh et al. 2011; Izquierdo and Clark 2012; Reyers et al. 2012a; Schröter et al. 2014b).

A first aim of this study was to review ES hotspot definitions and methods to spatially delineate hotspots and to classify the different approaches in order to distinguish the principle differences between them. We furthermore examined whether the reviewed studies indicate which policy purpose they intended to serve. A second aim was to apply and compare the outcome of these methods. Therefore, in a subsequent step we applied a selection of four hotspot delineation methods to a hypothetical ES conservation scenario which intended to prioritise areas to conserve 10 % of the total amount of each service. For this conservation scenario we used spatial models of five ESs, which have been developed for the county of Telemark in southern Norway (Schröter et al. 2014a). In order to critically appraise the hotspot approach we compared the outcomes of the four applied hotspot methods to the site prioritisation approach of Marxan for the same set of ESs for forest areas in Telemark. We compared all five approaches in terms of characteristics of selected sites, namely difference in spatial configuration (area size, location, and shape) and mean achievement of the ES conservation target.

### Review of ecosystem service hotspots

We reviewed ES hotspot definitions and delineation methods by means of a literature search. A Scopus search was performed on 22 May 2015. Search terms were adjusted until a pre-selection of studies dealing with spatial analysis of ES hotspots were all included in the search results. Title, abstract and keywords were searched for the terms “ecosystem” AND “services” AND (“hotspot*” OR “hot spot” AND “map*” OR “spatial” OR “overlap”). As earlier studies in the field did refer to ESs under the term landscape functions, a second search was done replacing the terms “ecosystem” and “services” by “landscape” and “functions”. The two searches were combined. A total of 158 studies were obtained after the initial search. Titles and abstracts were checked and only studies that performed an empirical spatial analysis on ES hotspots were selected. Some studies had done spatial analyses related to ES hotspots, but either generally defined hotspots as areas of importance for generating a service (Palomo et al. 2014), or related hotspots to spatial coincidence of landscape metrics, which were not clearly connected with ESs (Bryan et al. 2010). After excluding such studies, 23 papers were included in the review, dating from 2008 to 2015. Definitions and delineation methods were recorded, structured and classified. Through content analysis we assessed which potential policy purpose for their hotspot analysis the authors had indicated.

### Review results

**Fig. 1:**
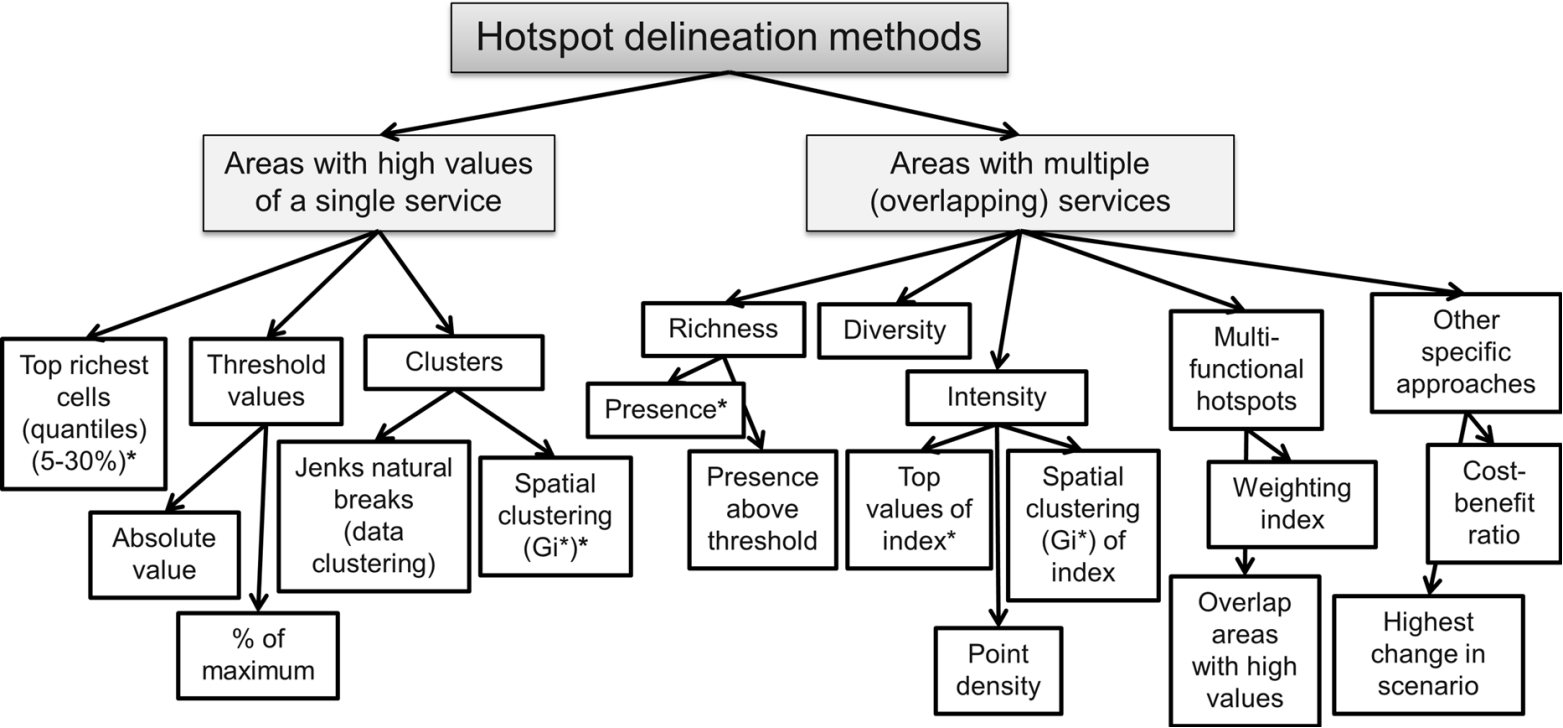
Classification of hotspot delineation methods. Methods with an *asterisk* were tested in this study

Two principal concepts to define hotspots were distinguished, which were each addressed by different delineation methods. Hotspots were defined in the reviewed papers either as areas with high values of one single ES or as areas containing multiple, overlapping ESs (Fig. 1).

The most common way to define an ES hotspot was in line with the definition of Egoh et al. (2008, p. 136), who defined hotspots as “areas which provide large proportions of a particular service”, where large proportion refers the upper range of service provision in an area. This approach was used in 13 of the 23 studies included in the review (Table 1). These studies often create hotspots based on a single service, but combine the different areas to define overall priority areas for conservation and management of services. While these studies used the same approach to define ES hotspots, the applied delineation methods differed. Three main delineation methods can be distinguished. First, a top richest cells (quantile) method divides high-to-low ranked grid cells with ES values into classes with an equal number of cells. According to this method the class with the highest values is chosen as a hotspot, while class definition ranged between 5 and 30 %, i.e. between the highest of 20 equally sized classes (vigintiles) and the top three deciles. Whether a top decile also accounts for exactly the top 10 % richest cells depends on ties (equal values of grid cells at the threshold between classes) (Eigenbrod et al. 2010). Second, a threshold method delineates a hotspot according to an expert-based biophysical threshold value of a particular ES, for example for the ES soil accumulation, a soil depth ≥0.8 m and ≥70 % litter cover in a specific case study (Egoh et al. 2008). This differs from the former approach as the threshold method does not consider the distribution of the ES over the grid cells. Third, cluster methods have been used to delineate hotspots with the help of Jenks natural breaks, where differences between classes are maximised according to clusters inherent in the data (Mitchell 1999). As a spatial clustering method, the G_i_^*^ statistic (Getis and Ord 1992) was used, which finds spatial clusters in the data to identify hotspots or coldspots (Mitchell 2005) (further explained below).

**Table 1:** Methods, policy purpose and reasoning, and number of ES considered in the reviewed studies

Hotspot method class	Study	Study area	Hotspot delineation method	Policy purpose and reasoning behind hotspot analysis	No. of ESs (no. of biodiversity layers)
Top richest cells (quantiles)	Eigenbrod et al. (2010)	England (Great Britain)	Richest 10, 20, 30 % of grid cells	Priority setting Congruence with biodiversity Methodological interest	2 (1)
	Bai et al. (2011)	Baiyangdian watershed (China)	Richest 10 % of grid cells	Priority setting/optimize conservation strategies Congruence with biodiversity	5 (1)
	García-Nieto et al. (2013)	Eight municipalities in Andalusia (Spain)	Richest 5 % of grid cells	Priority setting	6
	Wu et al. (2013)	Seven administrative units (northeast China)	Richest 10 % of grid cells	Priority setting (multiple services hotspots) for conservation/land management/planning	5
	Locatelli et al. (2014)	Costa Rica	Richest 25 % of grid cells	Priority setting/optimise conservation strategies Target management interventions	3 (1)
	Schulp et al. (2014)	European Union	Richest 25 % of grid cells	Assessment of importance of one single ES	1
	Rodríguez et al. (2015)	Colombia	Richest 10 % of grid cells	Priority setting Planning carbon and water resource management	5
Threshold value	Egoh et al. (2008)	South Africa	Service specific, expert opinion based threshold of an ES value^a^	Priority setting for conservation Support ecosystem management	5
	Egoh et al. (2009)	South Africa	Same as Egoh et al. (2008)	Priority setting for conservation Congruence with biodiversity	5 (1)
Jenks natural breaks	O’Farrell et al. (2010)	Succulent Karoo biome (South Africa)	Jenks natural breaks (top of three classes)	Priority setting for specific management	3
	Reyers et al. (2009)	Little Karoo (South Africa)	Jenks natural breaks (top of three classes)	Priority setting, conservation of ES	5
	Onaindia et al. (2013)	Urdaibai Biosphere Reserve (Spain)	Jenks natural breaks (top of three classes)	Priority setting for conservation Information for land management	2 (1)
Spatial clustering (${G_{i}^{*}}$)	Timilsina et al. (2013)	Florida (USA)	Getis-Ord ${G_{i}^{*}}$ statistic to identify clusters of plots with higher or lower carbon values	Priority setting Information for land management Determine drivers affecting hotspot patterns	1
Richness	Gos and Lavorel (2012)	Lautaret (France)	Presence of all (3) ES (preceding threshold analysis for determining areas of ES provision)	Congruence with biodiversity Information for management Methodological interest	3 (1)
Richness and Diversity	Plieninger et al. (2013)	Upper Lusatia Pond and Heath Landscapes Biosphere Reserve (Germany)	Areas of high intensity, richness and diversity of ES	Priority setting Identification of areas important for management	8
Intensity	Willaarts et al. (2012)	Sierra Norte de Sevilla (Spain)	Richest 1/3 quantile of grid cells of an overlap index	Priority setting (key provisioning areas) Provide information for integrated management	9
	Beverly et al. (2008)	Boreal and Foothills Natural Regions in west-central Alberta (Canada)	High point density of all services combined	Inform fire risk management to focus limited resources	9 (1)
	Queiroz et al. (2015)	Norrström drainage basin in south-central Sweden	High values of combined ES value maps that scored above average compared to the study area	Understand interaction patterns between ES	16
	Bagstad et al. (2015)	Pike–San Isabel National Forest in Colorado (U.S.A.)	Getis-Ord ${G_{i}^{*}}$ statistic to identify clusters of a summed value map of ESs	Assess synergies, trade-offs and conflicts with social values	4 (1)
Multi-functionality	Gimona and van der Horst (2007)	North-east Scotland (United Kingdom)	Areas scoring high for all three ESs in different weighing scenarios	Identify priority areas for conservation Include stakeholder preference in determining priority areas	2 (1)
	Willemen et al. (2010)	Gelderse Vallei (Netherlands)	Areas where combinations of ES lead to an increase in a specific ES compared to a region’s mean of this ES.	Support land use planning	7
Other specific approaches	Crossman and Bryan (2009)	Murray–Darling Basin (Australia)	Index weighting costs and benefits of ES restoration	Priority setting for restoration	4
	Forouzangohar et al. (2014)	Northern Victoria (Australia)	Positive change of 2 ES in a scenario analysis	Support land management and land use decisions	2

^a^Surface water supply: runoff ≥70 million m^3^. Water flow regulation: ≥30 % of total surface runoff. Soil retention: areas with severe erosion potential and vegetation/litter cover of at least 70 %. Soil accumulation: ≥0.8 m depth and a 70 % litter cover. Carbon storage: high (classified) = thicket, forest

Another principle approach of hotspot definition characterised hotspots as key areas providing more than one ES, a principle which was applied in different ways by 10 of the 23 studies. Four of these were based on aggregated ES value maps, i.e. summed and merged indices. These included an intensity approach, e.g. the highest quantile of a normalised multiple services index (“intensity”) (Willaarts et al. 2012), high point densities of multiple services (Beverly et al. 2008), summed values of multiple ES value maps where values are above average of the study area (Queiroz et al. 2015) and spatial clustering of an aggregated ES value map (Bagstad et al. 2015). Another type referred to the presence of several or all ESs included in an analysis (“richness”) (Gos and Lavorel 2012). In a particular case, Gos and Lavorel (2012) referred to ESs presence above a certain threshold. Others, however, refer to hotspots as areas that are either rich in different ESs (presence without a threshold) or show a high diversity of services (Plieninger et al. 2013). Two studies distinguished multifunctional areas as hotspots. Gimona and van der Horst (2007) delineated hotspots as areas where all considered ESs had high values. Willemen et al. (2010) delineated “multifunctional hotspots” as areas where combinations of ESs (called landscape functions) lead to a higher amount of a specific ES compared to a region’s mean of this ES.

Finally, two studies have defined hotspots in a way that specifically relates to their research interest, but all were related to the spatial congruence of two or more ES. Crossman and Bryan (2009) defined hotspots as areas with a high ratio between a multiple ES index and an index of opportunity costs of conservation. Forouzangohar et al. (2014) delineated areas as hotspots when both of the analysed services showed a positive change in a scenario analysis.

## Methods

### Case study area

**Fig. 2:**
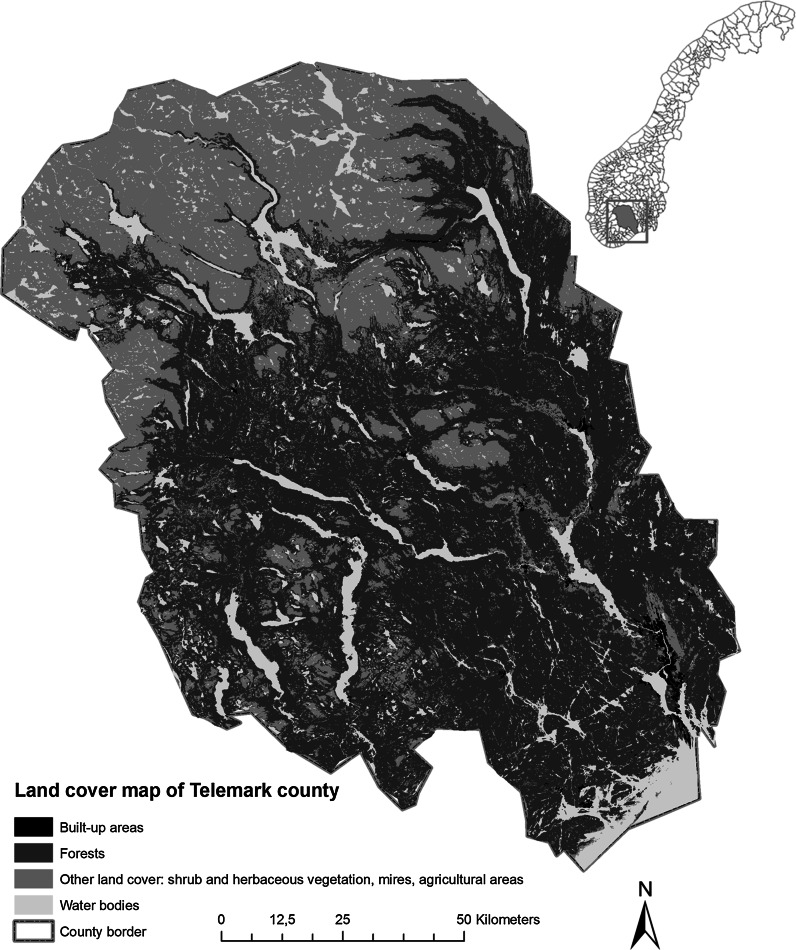
Simplified land cover map of the study area Telemark and its location in Norway. Data source: Norwegian Mapping authority (AR 50 dataset)

Telemark is a county in southern Norway with an area of 15,300 km^2^ and a population of about 170,000 (SSB 2012). The climate varies across the region with temperate conditions in the south-east (Skien, average temperature January −4.0 °C, July 16.0 °C, 855 mm annual precipitation) and alpine conditions in the north-west (Vinje, January −9.0 °C, July 11.0 °C, 1035 mm) (Meteorological Institute 2012). The forest landscape is characterized by coniferous and boreal deciduous forest (Moen 1999). A land cover map of the study area is shown in Fig. 2. As forest field mapping lacks for a small south-eastern part of the county (NFLI 2010), we excluded this area for the analysis.

### Spatial models of ecosystem services

Five key ESs for Telemark, for which spatial biophysical models have been developed (Schröter et al. 2014a), were included in the analysis: carbon storage, carbon sequestration, snow slide prevention, recreational hiking and existence of wilderness-like areas. We used ES flow models for this current analysis, i.e. models reflecting the actual use of ESs. The selected ESs are conservation-compatible (Chan et al. 2011). This means that the occurrence of the service could reasonably be taken into account as an (additional) argument for conservation, and conservation would not restrict their use. Many provisioning services, such as timber production require management and (more or less intensive) extraction, and their use would normally be restricted in conservation areas, i.e. they are often not compatible with conservation. Regulating services, on the other hand, can be considered conservation-compatible if their use does not require large human interventions. Carbon sequestration and storage and snow slide prevention are examples. Cultural services can be considered conservation-compatible if their use does not conflict with conservation objectives. In the case of Norway, protected areas are often open for low-impact recreational hiking. Other cultural services, however, would conflict with conservation, such as building infrastructure for holiday cabins.

We shortly describe indicators and main inputs of the models here; detailed methods for the development of the spatial ES models can be found in Schröter et al. (2014a). Three regulating services were included (carbon storage, carbon sequestration, snow slide prevention). Carbon storage (Mg C ha^−1^) was based on field data on above- and belowground carbon stocks. Carbon sequestration (Mg C ha^−1^ year^−1^) was modelled as the difference between net primary production and soil respiration. Snow slide prevention by forests mediates flows in a beneficial way and reduces the risk of avalanches. This service was delineated as forest areas on snow slide release areas, whenever infrastructure was present in the respective propagation areas (indicated by presence only). The two cultural services were recreational hiking and existence value. For recreational hiking we built an index containing density of hiking paths in an area weighted by potential users in a defined surrounding. This index reflected both accessibility to hiking areas and potential use by people. Existence of wilderness-like areas was modelled as areas with a distance of more than 1 km from large infrastructure (e.g., roads, power lines) (indicated by presence only). The presence indicator for this ES merely reflects the existence of such areas, but not an active use. This service stands for the value that many people hold for the pure existence of certain ecosystems (Krutilla 1967; Noss 1991; Reyers et al. 2012b). Both the snow slide prevention model and the existence of wilderness-like area model are constructed with a presence-absence logic. While they give an indication of the spatial distribution of the ES, they do not assign different biophysical values to the site, but rather a “1” for presence and a “0” for absence.

### Testing different hotspot delineation methods

We applied and compared four different hotspot delineation methods for a conservation scenario for the five ESs for forest areas of Telemark, in which we assumed a conservation target of 10 % of the biophysical amount of each ES. This target is arbitrary, but ensured comparability among the approaches as all methods were adapted so that the hotspots of each ES accounted for approximately the same biophysical amount. All spatial analyses were done in ArcMap 10 (ESRI).

We selected two hotspot delineation methods that are based on single ESs and subsequently combined the prioritised areas of each ES, and two methods that were based on multiple services. The selected delineation methods to create hotspot maps were the top richest cells approach, spatial clustering (G_i_^*^ statistic), intensity and richness.

The top richest cells approach was the most commonly used approach and was thus also considered in our study. However, as two of the ES maps were presence data only (snow slide prevention, existence value), a top richest class could not be determined and these two ESs were excluded. Spatial clustering was chosen as this approach is an established method to determine hotspots in geographic information science, while it has not often been applied to ESs yet. As it is based on metric data, the presence data ESs were excluded here as well. A simple richness approach was taken that does, in contrast to the more elaborate approach in Gos and Lavorel (2012), not systematically search for thresholds first. The intensity approach was chosen as standardisation and aggregation of ES is a widespread, practical and straightforward index in studies on multiple ESs (Maes et al. 2012b; Schneiders et al. 2012; Pan et al. 2013). The threshold value approach was not applicable as expert based thresholds for ‘high’ levels of services were not available and also would not allow to pursue a specific overall target for ES conservation. The spatial delineation of top richest cells and Jenks natural breaks does not differ when a fixed amount in the top class is pursued. As the number of classes in the Jenks natural breaks approach would be adapted until the amount in the top class equals the amount in the top richest cells approach. Other approaches found in the review were either too specific in their respective purpose or not applicable to the data sets (e.g. point data based indices).

### Hotspot maps

The four hotspot maps were created as follows. Data preparation steps for each method can be seen in Table 2.

**Table 2:**
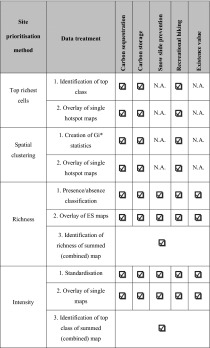
Data preparation for each hotspot delineation method

*NA* not applicable

#### Top richest cells

According to the top richest cells approach, we sorted all grid cells with descending values and iteratively adapted a top class and calculated the sum of cells in this class until the sum amounted to approximately 10 % of each ES. This iterative testing involved choosing a top quantile and when the total amount covered in this class was higher (or lower) than the 10 % target, choosing a smaller (or larger) top class. This process aimed at minimising the difference between the sum of grid cells above a threshold value and the 10 % target. In a next step, the three ES hotspot maps were merged to one single map.

#### Spatial clustering

Spatial clustering for finding hotspots with the help of the G_i_^*^ statistic identifies high concentrations of pixels with high values within a specified distance. We followed a stepwise approach (Timilsina et al. 2013; ESRI 2014). First, for each of the three ES separately, we determined the average distance of each grid cell containing the ES to its nearest neighbour also containing the ES. We then determined the distance band from each cell that maximised spatial autocorrelation. We calculated the z-score of Global Moran’s I with the distance band equal to the average distance to the nearest neighbour, and increased this iteratively by 1 km until the z-score reached a maximum. This distance band was used for the G_i_^*^ statistic in ArcMap 10 (Mitchell 2005) according to

1 $$G_{i}^{*} (d) = \frac{{\mathop \sum \nolimits_{j} w_{ij} (d)x_{j} }}{{\mathop \sum \nolimits_{j} x_{j} }}$$

where *G*_i_^*^ (*d*) is the statistic calculated for each grid cell, *d* is the distance band for finding neighbours as determined in the precedent step, *w*_*ij*_ is a binary weight (1 for cells within *d*, 0 for cells outside *d*), *x*_*j*_ is the ES value for each of the five ES models.

We calculated a Z-score for testing the significance of the G_i_^*^ statistic for each cell according to

2 $$Z\left( {G_{i}^{*} } \right) = \frac{{G_{i}^{*} - E(G_{i}^{*} )}}{{\sqrt {{\text{Var}}(G_{i}^{*} )} }}$$

3 $$E\left( {G_{i}^{*} } \right) = \frac{{\mathop \sum \nolimits_{j} w_{ij} (d)}}{n - 1}$$

where *E*(*G*_i_^*^) is the expected *G*_i_^*^ value for random distribution and *n* is the number of grid cells. We then ranked cells from high to low Z-scores and iteratively selected the top cells until the sum of grid values corresponded to the 10 % target. This ensured that cells within the most significant clusters were included as hotspots. Here, as well, iterative testing aimed at minimising the difference to the 10 % target. All three ES hotspot maps were merged.

#### Intensity

For the intensity hotspot, all spatial models of ES were standardised (0–100) by subtracting from each cell the minimum value of each ES and dividing the difference by the range of each ES, and multiplying this ratio by 100:

4 $$x_{{j_{s} }} = \frac{{x_{j} - { \hbox{min} }(x_{j} )}}{{{\text{max(}}x_{j} ) - {\text{min(}}x_{j} )}}{ \times }\,100$$

where *x*_*js*_ is the standardised ES value of cell *j*. All five standardised maps were given equal weights and added to one ES index map (Maes et al. 2012b; Willaarts et al. 2012):

5 $$x_{{j_{I} }} = w{ \times }(x_{{j_{ESi} }} )$$

where *x*_*jI*_ is the index value of cell *j*, *w* = 0.2, *x*_*jESi*_ is the value of *ES*_*i*_ (i = 1,…,5). In absence of other knowledge and for the sake of simplicity, all ES were thus assumed to be equally important. In accordance with the method used in Willaarts et al. (2012), quantiles were used to determine the top class that forms the hotspot. In contrast to the former hotspot delineation methods, the intensity method accounts for ES bundles and not for a combination of single ESs. Thus, the size of the top quantile was iteratively adapted until the mean target achievement of all five ESs approached 10 %. This iterative testing involved choosing a starting top quantile and when the total amount covered in this class was higher (lower) than the 10 % target, choosing a smaller (larger) top quantile. However, as two of the five ESs had a standard (presence) value of 1, the relative importance of those two services within the hotspot increased when the data was classified into a higher number of quantiles, while the biophysical amount of the three other ESs decreased remarkably. We thus decided to stop the iterative search process for the top quantile in order to prevent a selection bias towards two ESs and to consider all five ESs. The iterative search was stopped at 20 classes, i.e. the top quantile of 20 quantiles represented the hotspot.

#### Richness

For the richness method we merged the distributions of all five spatial ESs models (with a presence value of 1 for each model), which resulted in a raster grid with values of 0 (no ES present) to 5 (all five ESs present). We then analysed, which ES richness, i.e. which number of present ESs, was required to build a hotspot that most closely approached the 10 % target as a mean for target achievement for all services. Transforming metric scale data to a presence-absence logic implies a simplification of the importance of each pixel as it neglects the amount of service provided per pixel. As such, a shift of important areas could be expected. However, as we also calculated the total amount of ESs covered by each of the hotspots, comparability is given. We will discuss this in more detail in the discussion section.

### Heuristic site prioritisation with Marxan

Marxan is a conservation site selection software building on an optimisation algorithm which incorporates key principles of systematic conservation planning (Margules and Sarkar 2007). These principles include comprehensiveness, i.e. reaching multiple targets, cost-effectiveness, i.e. finding solutions for the least possible cost, and compactness, which implies a low edge to area ratio (Wilson et al. 2010). Marxan (version 2.43) works with a heuristic optimisation algorithm with the help of simulated annealing (Ball et al. 2009). The software aims to minimise an objective function containing the sum of opportunity costs of conservation, represented by the costs of selected planning units and the boundary length of the reserve system. The objective function contains penalties for not meeting conservation targets as well as for breaching a given cost threshold (Game and Grantham 2008). Conservation targets are set as a proportion of the total amount of each feature in a study area. Thereby, the software allows to integrate both (binary) presence/absence data of a conservation feature and metric biophysical data of different kinds into the same decision problem. The software requires a series of inputs. Conservation targets were set at 10 % for the total amount of each ES in the study area. We divided the forest area into 241,013 quadratic planning units of 4 ha size each. This resolution was chosen as it was manageable for the software in terms of time and computing capacity (Alidina et al. 2010), while at the same time it was high enough to cover spatial heterogeneity in an adequate way. For each planning unit we calculated the amount of each ES contained in that unit. For the sake of comparability with the hotspot approach, we decided not to include site specific opportunity costs of conservation, which would have had an influence on the site selection. We therefore assigned a standard opportunity cost of 1 to each planning unit. Marxan requires a number of parameters to be set (see ESM of Appendix 1 for details). The boundary length modifier was set according to methods described in Game and Grantham (2008) in order to guide the software to select a compact, spatially coherent reserve network. A feature penalty factor was set in order to reach a high target achievement in each scenario according to the iterative procedure described in Game and Grantham (2008). Marxan was run 100 times with these parameters. The map of selected sites was produced by ranking all planning units according to the number of runs in which they have been selected (selection frequency). The selection frequency that led to a selection of sites that most closely approached the mean 10 % target for all ES was chosen. Two Marxan analyses were performed in order to compare the outcome to the different hotspot delineation methods. One analysis included the three ES measured in metric data (carbon sequestration, carbon storage and recreational hiking), and one included all five ESs.

### Comparison of selected areas (hotspots, Marxan)

Each of the four hotspot delineation methods and the selected sites of the two Marxan analyses yielded a spatial prioritisation of areas. For comparison, we recorded the area size and calculated the edge-to-area ratio (where edge is the sum of the boundary lengths of all selected sites), the target achievement for each ES and the mean target achievement for all prioritized areas. We also calculated the ratio of area to mean target achievement in order to compare the different methods. Pairwise, we tested the agreement of spatial configuration between all maps with Cohen’s Kappa. For this purpose, all maps were defined as presence (1, cell selected) and absence (0, cell not selected). Each of the 787,396 cells were assigned presence and absence values for each map.

## Results

### Selected areas for hotspots and Marxan

**Fig. 3:**
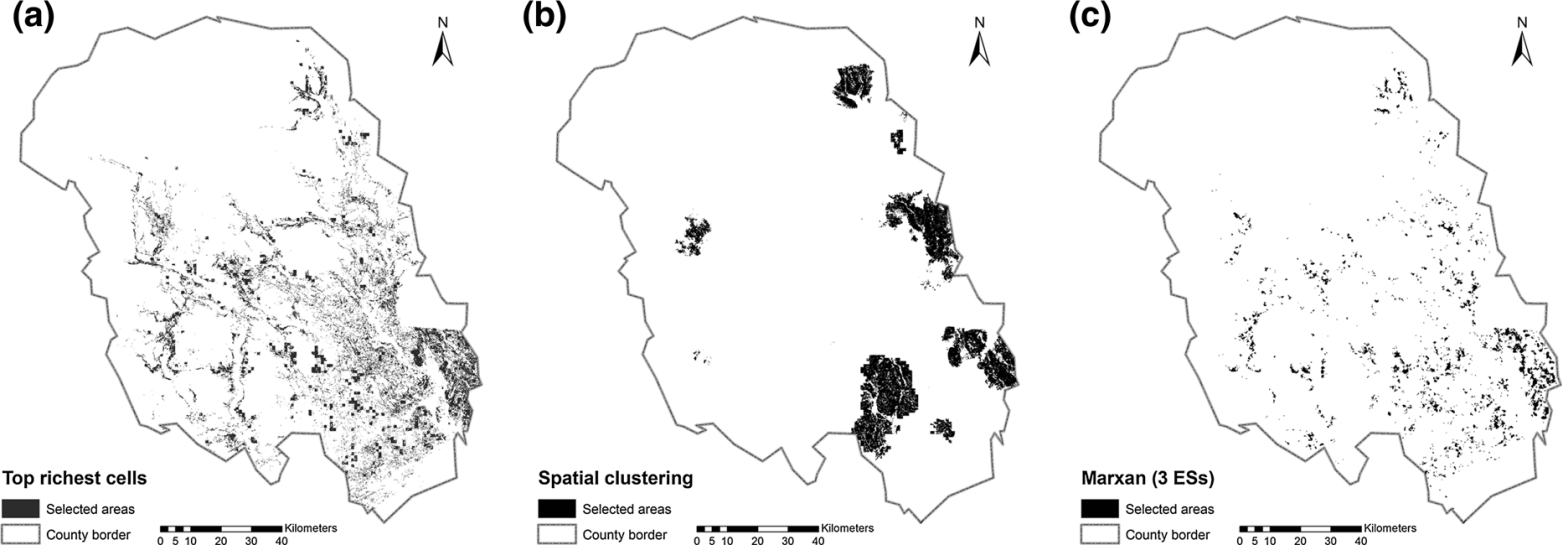
Maps of areas selected as hotspots according to the top richest cell approach (**a**), spatial clustering (**b**), Marxan (three ecosystem services) (**c**)

Maps for the top richest cells, spatial clustering and the Marxan result for three ESs with metric data (carbon sequestration, carbon storage, recreational hiking) are presented in Fig. 3. Figure 3a shows all areas that are in the top quantile for at least one ES (top richest cells). It is inherent to the method that, because the hotspots for each ES do not completely overlap, the total selected areas for the three ESs is relatively large and dispersed, which we discuss in further detail below. The respective classes were 16 quantiles (carbon sequestration), nine quantiles (carbon storage) and 209 quantiles (recreational hiking). This large number of quantiles was due to the fact that for recreational hiking a relatively few number of cells had extraordinary high index values, representing 10 % of the total amount of the services. Figure 3b shows the spatial clustering outcome, which is also constructed as the sum of three hotspots. As this method searches for clusters within the data, the outcome appears less dispersed than the one of the top richest cells method. Figure 3c show the result of Marxan for the three ESs. A minimum selection frequency of 23 (of 100 runs) was determined as the threshold that led to an area large enough to achieve a mean of approximately 10 % of the ES target. There was an overall tendency of areas to be selected in the east and south of the county. This was mainly due to high abundance of high value cells of the recreational hiking service, which contains information about people living in proximity. Furthermore, this area contains highly productive forest, leading to relatively high abundance of cells with high carbon sequestration and storage values.

**Fig. 4:**
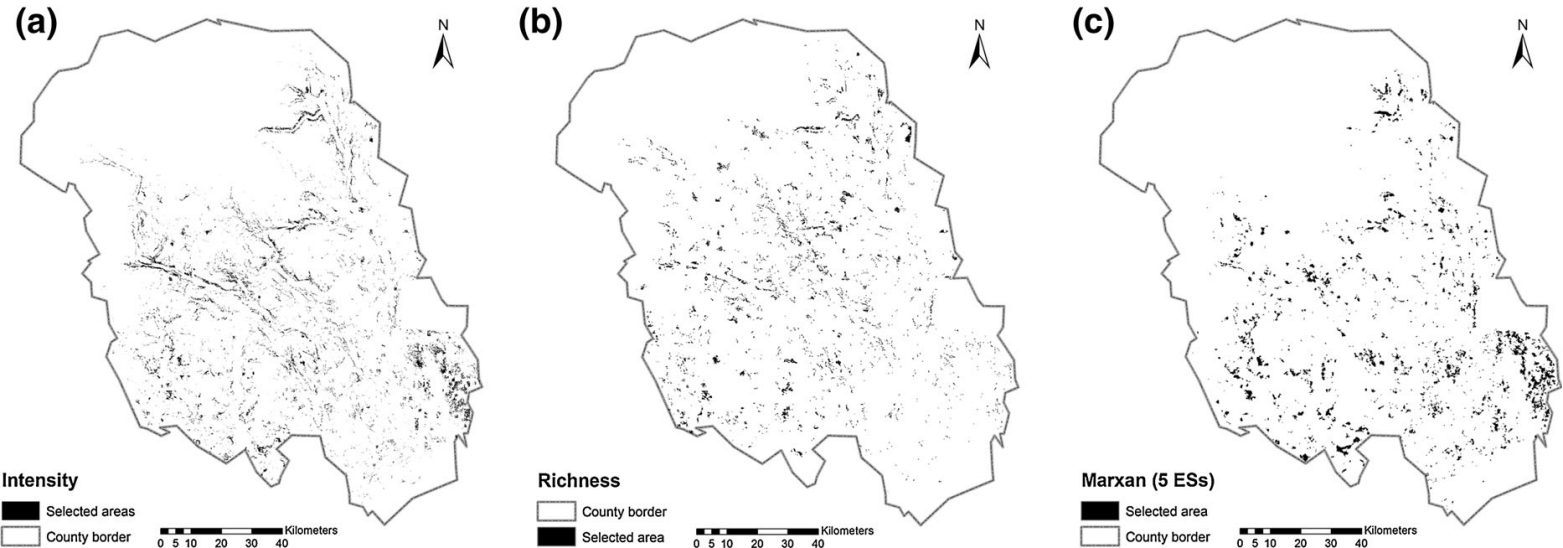
Maps of areas selected as hotspots according to the intensity approach (**a**), richness approach (**b**) and Marxan (five ecosystem services) (**c**)

Figure 4 shows the outcomes of the hotspot delineation methods and Marxan for all five ESs. Figure 4a) shows the highest of 20 classes of the sum of the standardised ES models (intensity approach). The result is more scattered across the study area and a considerable smaller total area was selected as the method does consider multiplicity of ESs and consequently chooses areas were ESs overlap. Figure 4b shows the result of the richness approach, which depicts areas with an overlap of at least four of the five ESs. This number was required to cover approximately 10 % of each ES (see also Table 4 for statistics on conservation results). Figure 4c) shows the results of the site selection of Marxan. A minimum selection frequency of 22 (of 100 runs) was determined as the threshold that led to an area large enough to achieve a mean of approximately 10 % of the ES target. The result is several clumped areas spread over the study area.

### Spatial agreement of selected areas

Spatial configurations of the results according to the four hotspot methods and Marxan differed considerably. All results for the pairwise comparisons (Table 3) are at the lower end of the scale of the Kappa statistic, of which values close to 1 would indicate almost perfect agreement (Landis and Koch 1977). Pairwise comparisons showed slight agreement for four of the six comparisons. Fair agreement was observed between Marxan and the top richest cells approach as well as between Marxan and intensity.

**Table 3:**
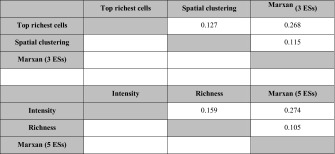
Pairwise agreement between selected areas measured with Cohen’s Kappa (K)

Values between 0 and 0.20 indicate slight agreement, and values between 0.21 and 0.40 fair agreement (Landis and Koch 1977)

All values significant at p < 0.001

### Comparison of aggregated target achievements and selected areas

Target achievement for single ESs differed depending on the applied method (Table 4). For instance, the intensity method exceedingly selected the ES snow slide prevention (58.7 %). This was partly due to the construction of this model as a presence-absence model (0–1 binary scale). As such, all areas containing this ES had a relatively high value, and thus a higher chance to be selected from the summed standardised intensity map. Furthermore, these areas more often overlapped with highly productive forest, which increased their chance to also have higher than average values for carbon sequestration and storage. With Marxan targets were achieved approximately even around 10 % (low standard deviation and low coefficient of variation, Table 4). Mean target achievement was also close to the 10 % target for richness. Mean target achievement was considerably higher for the top richest cells approach and spatial clustering. This was partly because these methods were first based on single ESs and were merged in a subsequent step. As the hotspots for all single ESs only partly overlapped, the total area of the combined single ES hotspot maps was larger. When an ES was present in areas that formed a hotspot of another ES, these additional selected and thus conserved ESs could be viewed as side benefits.

**Table 4:** Comparison of selected areas for the four hotspot methods and Marxan

	Area in km^2^	Mean ES target achievement in % (σ/CV)	Area/mean ES target achievement ratio	Edge/area ratio	Target achievement single ecosystem services				
					Carbon sequestration	Carbon storage	Snow slide prevention	Recreational hiking	Existence value
Top richest cells (3 ESs)	1238	28.7 (5.3/0.3)	4305	15.8	22.6	28.0	N.A.	35.6	N.A.
Spatial clustering (3 ESs)	1028	20.8 (5.4/0.3)	4934	4.4	17.7	16.4	N.A.	28.5	N.A.
Marxan (3 ESs)	354	9.6 (3.0/0.3)	3686	8.4	7.8	7.1	N.A.	13.9	N.A.
Intensity (5 ESs)	409	18.3 (20.6/1.1)	2237	22.1	7.6	7.2	58.7	15.0	2.9
Richness (5 ESs)	290	7.7 (5.5/0.7)	3773	12.8	3.5	2.7	14.5	3.4	14.3
Marxan (5 ESs)	445	10.7 (2.3/0.2)	4144	8.5	8.5	7.8	10.7	13.2	13.5

Table 4 summarises characteristics of the selected areas for the four hotspot methods and the two Marxan outcomes. For three ESs, the sum of selected area was smallest for Marxan, and highest for the top richest cells approach. For five ESs the area was smallest for the richness approach and highest for Marxan. Marked differences in selected areas and mean target achievements (9.6–28.7 % for 3 ESs and 7.7–18.3 % for 5 ESs) made comparison between approaches challenging. We thus calculated the ratio of area to mean target achievement as an indicator of how efficiently land is selected in order to achieve targets. This indicator was lowest for Marxan (3 ESs) and the intensity approach (5 ESs), and highest for spatial clustering (3 ESs) and Marxan (5 ESs). As expected, the intensity approach scores best in conserving relatively high amounts of ESs per land area, which leads to a low area-target achievement ratio. Spatial clustering through the G_i_^*^ statistic is constructed as such that it also includes cells that have a low value, but are in the vicinity of neighbours with high values. By doing this, spatial clustering needs more area per unit target achievement, but achieves a low edge-to-area ratio. The top richest cells approach, on the other hand, selects high value cells that can, depending on the respective ES, be scattered across the landscape. This leads to a higher edge-to-area ratio. This edge-to-area-ratio is highest for the top richest cells approach (3 ESs) and the intensity approach (5 ESs).

## Discussion

### What is an ecosystem service hotspot?

Despite the ample use of the term hotspot within the ES literature, we observed that within the reviewed studies there was no consensus on what a hotspot is. There was, however, a tendency to characterise ES hotspots as areas of high values of single services, which is in line with the definition of one of the first studies published on that topic (Egoh et al. 2008). While 13 of the 23 reviewed studies used the same principle construction of a hotspot, a variety of methods to delineate the hotspot was observed. The lack of consensus and an exploring, occasionally pragmatic way of method development could be seen as characteristic for the current advancement in the relatively young scientific field dealing with ESs (Jacobs et al. 2013; Schröter et al. 2014c). We discuss three aspects to further develop the notion of ES hotspots in the future, namely the inclusion of threats, ES demand and a distinction of conservation-compatible ESs.

Interestingly, the definitions currently applied in ES hotspot mapping differ from the earlier established notion of a biodiversity hotspot, which has been defined as an area of both high biodiversity and high level of threat, i.e. probability of destructive ecosystem exploitation (Myers 1988, 1990; Mittermeier et al. 1998; Myers et al. 2000). Being one of the first studies to map ES hotspots, Egoh et al. (2008, p. 136) even explicitly state that they “do not include measures of threat”. Later studies also did not include threat in the definition and delineation of hotspots. One way to include threat in a future study for Telemark could be to consider accessibility of forest areas and profitability of forest exploitation as an indicator of threat (Naidoo et al. 2006). In the case of Telemark, clear-cutting can be regarded as having detrimental effects on a number of ESs and biodiversity (Schröter et al. 2014b).

Furthermore, targets for services represented within hotspots need to be formulated, i.e. a level of services that is considered particularly important from a societal point of view needs to be determined (Mastrangelo et al. 2014). Target setting of ESs for the purpose of conservation is not common practice yet (Luck et al. 2012b). New insights from research on defining demand for ESs (Wolff et al. 2015) could be integrated to formulate targets for absolute amounts of ESs. Demand could for instance relate to absolute amounts of services used in an area (Burkhard et al. 2014) or to preferences and desires regarding services (Wolff et al. 2015).

In order to meaningfully represent multiple ESs in a hotspot for the purpose of site selection for conservation, we argue that only those ESs that do not require substantial human interventions during management and harvest should be considered due to trade-offs that can occur between ESs. Many regulating and cultural ESs either show none or synergistic interactions with one another (Bennett et al. 2009) and can meaningfully be represented in a hotspot. Extractive provisioning services, such as clear-cutting timber harvest, however, impede other services such as carbon sequestration or hiking. Knowledge on effects of the use of one ES on another ES is still missing. We observed that the reviewed studies often have chosen to determine hotspots with multiple regulating and cultural ESs, which presumably have no or synergistic interactions with one another (e.g., Egoh et al. 2008; Bai et al. 2011; Locatelli et al. 2014). Such areas could meaningfully be considered as priority sites for conservation of ESs next to biodiversity. When multiple potentially conflicting ESs are considered together, for instance, timber harvest, forage or hydropower next to cultural and regulating ES (Willaarts et al. 2012; García-Nieto et al. 2013; Wu et al. 2013), the resulting areas are probably more useful to determine ‘conflict spots’ or ‘coldspots’ (sensu Willemen et al. 2010), which would require integrated management to reduce specific known trade-offs and interest conflicts.

### When to choose which prioritisation method?

We found no clear link between distinct hotspot methods and specific policy purposes in our review. Most reviewed articles state generic and relatively similar purposes for applying hotspot methods, irrespective of the method used. Priority setting was most commonly stated (16 out of 23), along with informing or supporting (land) management and planning (9 out of 23) (Table 1). In principle, all hotspot methods fit these broad policy purposes, as assessing areas of high value is their core methodological purpose. Only in a few cases a specific policy purpose was stated, such as matching ES hotspots with hotspots of social value to assess synergies, trade-offs and conflicts (Bagstad et al. 2015) and informing fire risk management to focus limited resources (Beverly et al. 2008).

An important aspect to consider when choosing for either a hotspot method or a heuristic site prioritisation approach, is whether the intensity of ESs per unit land area matters for its long-term provision. From an ecological point of view, more knowledge is required on the functional traits underlying ESs as well as the spatial and temporal scales influencing ESs (Kremen 2005). From a human benefit point of view, whether intensity matters or not depends on the respective ES. For recreational hiking, one might be interested in including sites of relatively high value in a reserve and for existence of wilderness-like areas, a large, remaining area might be more valuable and preferable to include. For such ESs, hotspot methods might be more informative for decision making than an analysis with Marxan. For other ESs, however, such as carbon storage and sequestration, the total amount of conserved ES matters much more than the configuration of the selected areas. Contrary to being selected in a hotspot, such services could be spread across many connected sites containing small to medium amount of the ES. We have demonstrated that also hotspot methods can lead to considerable scattering. If ecological characteristics, such as landscape connectivity are important for the provision of services (Mitchell et al. 2015), then these hotspot methods might not be suitable for finding areas for conservation of these services. The spatial clustering hotspot method generates larger clustered areas than other hotspot methods and would therefore be most relevant if for example a single protected area or recreational area would need to be defined. Such large areas, connected throughout the landscape might be recommendable for some ESs, such as recreational hiking, which could lose a considerable part of their value if neighbouring areas are not conserved.

The principal difference between using a single or multiple ES for delineating hotspots has consequences for taking into account the concept of landscape multi-functionality (de Groot 2006; Gimona and van der Horst 2007; Mastrangelo et al. 2014), when prioritising a site for a specific policy purpose. In particular, the inclusion of cultural ESs can be regarded as a representation of different types of values. The simultaneous inclusion of different social and ethical values which are reflected by, for instance, cultural ES (Chan et al. 2012a, b; Luck et al. 2012a; Schröter et al. 2014c) might be better supported by the intensity and richness hotspot methods. To actually consider multi-functionality when applying the richness approach, only areas above a certain threshold should be included in order to prevent the inclusion of areas containing only marginal amounts of one or several ES. Such thresholds have been shown to influence the magnitude of overlap between ES (Anderson et al. 2009; Gos and Lavorel 2012). Defining and testing such thresholds before applying the richness approach was out of the scope of this study. Hotspot delineation according to methods that concentrate on one particular ES (top richest cells, thresholds, Jenks natural breaks, spatial clustering), merge areas that contain at least one ES. Such methods might in the first place prioritise areas for specific management actions towards one particular ES (O’Farrell et al. 2010; Locatelli et al. 2014). These studies, however, sometimes also consider multi-functionality by determining priority areas as overlaps between hotspots of single ES (Egoh et al. 2008; Bai et al. 2011; Wu et al. 2013).

### Differences in spatial configuration of hotspots and Marxan

We found marked differences in spatial configuration of selected areas depending on the hotspot method applied for the five ESs in Telemark’s forest areas. Kappa statistics for pairwise agreement of prioritised areas showed only slight to fair agreement and were at the lower end of the scale. These findings are important to consider for future studies on the spatial synergies among ESs and between ESs and biodiversity. If even hotspot methods following the same principle differ strongly in terms of spatial configuration of prioritised areas, then results should be carefully interpreted. We have also shown that areas prioritised by hotspot methods were different in terms of spatial configuration compared to more complex spatial prioritisation methods as used in Marxan. Depending on the purpose of the area selection, the use of Marxan might have advantages compared to the use of hotspots, which we discuss below.

We also found that, when applying the different hotspot methods, the outcomes differed strongly in terms of the total amount of ES provided in these areas (Fig. 3). and studies on conservation of ESs have to rely on assumptions and expert judgements when determining targets (Chan et al. 2006; Egoh et al. 2010; Chan et al. 2011; Izquierdo and Clark 2012; Schröter et al. 2014b). The hotspot studies we reviewed did not include explicit quantitative targets for ESs, but do however, implicitly set targets for a prioritised area when choosing a top quantile of different sizes (e.g. 5–30 %). Striving for explicit targets of ESs might, however, be more consistent with the current practice in conservation planning (Carwardine et al. 2009) than spatially determining hotspots which lead, depending on the method, to differing amounts of ESs on the selected sites. The difference in total ES quantities can be attributed particularly to skewness and spatial distribution of the data. The amount of ESs held in a top quantile strongly depends on skewness. In case of a negative skew (left-skewed distribution), a fixed proportion of top richest cells would contain a high total amount of ESs, while in case of a positive skew (right skewed distribution), the top richest cells would contain a lower amount. Spatial distribution of multiple ESs and the relation to each other also has an influence of the total amount of ESs included in a hotspot. This holds, for instance, for the richness approach, where the total quantitative sum of ES in the selected areas depends very much on overlaps between different ES. Overlapping areas can contain differing amounts of ES. Similarly, when determining a top class of a standardised sum of ES, as is done in the intensity approach, the spatial distribution of each single service and the location to each other determines the amount of ES present in the selected areas. Furthermore, constructing aggregated indices as the basis for the intensity approach is subject to weighting different ESs against each other. In this study, for simplicity reasons, we have assumed equal weighting. Gimona and van der Horst (2007), however, have shown how different weights influence the location of hotspots and suggest to combine differently weighted indices for determining areas that show high values regardless of the weights they applied (multifunctional hotspots).

In our study we attempted to combine explicit targets (10 % of biophysical ES amount) with the application of hotspots and Marxan. Mean target achievements differed, ranging from underachievement (7.7 %, richness approach) to strong overachievement (28.7 %, top richest cells). Especially those methods that select hotspots of single ES resulted in a high amount of side-benefits. This strong difference in both total amounts of ESs and in selected areas restricts the comparability of the spatial configuration of the outcomes, but substantiates the observation of notable differences in the approaches. It has been shown that changing targets for ESs influences size and spatial configuration of prioritised areas (Egoh et al. 2011). An uncertainty analysis in a future study could thus test to what extent the changing targets effect the differences between spatial configuration change of hotspots and Marxan.

### Criteria for site prioritisation in accordance with principles of conservation planning

The results presented here all prioritise areas for the purpose of conservation based on ES provision. Our approach should, however, be understood as a test of methods instead of as providing concrete suggestions for the location of reserves. First of all, the analysis is based on ESs only and does not include habitats of specific species or specific vegetation types which may be of high relevance for conservation. Hence, the biodiversity value of the areas is not considered in the ES-based selection approach. Biodiversity hotspots could, for instance be considered next to ES hotspots. Within the process of systematic conservation planning (Margules and Pressey 2000), site prioritisation should take into account both biodiversity and ESs, for which approaches have been tested in recent studies (Chan et al. 2011; Egoh et al. 2014; Schröter et al. 2014b). We discuss three criteria that are considered important for site prioritisation, namely comprehensiveness, compactness and cost-effectiveness (Possingham et al. 2006; Wilson et al. 2010).

The first criterion, comprehensiveness, refers to adequately meeting conservation targets (Wilson et al. 2009). Methods that are based on single ES overachieved targets when they were overlapped afterwards, as sites selected as hotspot areas for one service also provide other ESs. These methods are thus prone to selecting more areas than needed to achieve a target. In decision making, a more stringent selection of areas might still be needed if the conservation budget is not sufficient to conserve all sites. On the other hand, for methods that incorporate multiple ESs at a time, it depends on the overlap between ES and on the distribution of values whether some ESs are overrepresented Marxan contains comprehensiveness as one important factor in its objective function (Ball et al. 2009). While the software can be steered so that single solutions approximately reach the targets (Fischer et al. 2010), the approach we have taken here is based on selection frequencies, which can be considered as an indicator of how important a particular planning unit is (Possingham et al. 2010). Some ES targets were slightly overachieved, while others were slightly underachieved (Table 4). The second criterion, compactness, refers to a reserve system with a low edge-to-area ratio (Wilson et al. 2010). This indicator was lowest for the spatial clustering method, which selected compact, clustered sites including both high and low values within a certain neighbourhood. One disadvantage of this approach is that cells containing high amounts of ES are outside the selected clusters (Timilsina et al. 2013). Compactness is one of the objectives of Marxan and as such the edge-to-area ratio of the outcome of Marxan is relatively low, despite being considerably higher than that of the spatial clustering. All other approaches, in particular the intensity approach, selected many small, isolated sites. This led to a comparably high edge-to-area ratio.

The third criterion, cost-effectiveness, refers to reaching a specific conservation target for the least possible conservation cost (Naidoo et al. 2006). In this study we took the ratio of land area selected per mean target achievement as a parsimonious indicator for cost-effectiveness of selected areas. Methods that consider multiple ESs at a time (intensity and richness) need the least area per mean target achievement, followed by the outcome of Marxan. Spatial clustering, which selects cells with a low amount of ESs in proximity to cells with high amounts, showed the highest ratio of land to target achievement.

## Conclusion

Currently no consensus exists on how to define an ES hotspot. We found two principally different approaches, which either consider an ES hotspot as areas with a relatively high amount of one single ES or as areas containing multiple ESs. When applied to the case of five regulating and cultural ESs for Telemark, hotspot delineation methods differed strongly in terms of spatial configuration and amount of ESs covered by these areas. We found that a recurring aim of hotspots is to inform land use decisions through site prioritisation. The marked difference in spatial configuration among hotspot methods shows, however, that there are large uncertainties involved in site prioritisation, as different methods yield different results. The difference in spatial configuration can also have consequences for studies that analyse the spatial co-occurrence of hotspots of multiple ESs and of ES hotspots and biodiversity. While determining hotspots according to one approach might lead to high degrees of spatial overlap with another ES or biodiversity, other delineation methods might lead to considerably lower degrees of overlap.

We also found that setting specific targets for ES conservation was not common in the delineation of hotspots. Defining a hotspot as the highest of several classes of a dataset for a specific ES, as is common practice, can lead to very different amounts of ESs included in a selected sites depending on the method used. In an attempt to ensure comparability between the approaches we have defined arbitrary but specific targets for ESs, but also found considerable challenges in approximately reaching these targets.

We compared outcomes of hotspot methods to outcomes of the conservation software Marxan. While some hotspot methods score better than Marxan in terms of either comprehensiveness, compactness or cost-effectiveness, Marxan is able to consider these three criteria simultaneously and thus could be preferred over hotspots to select sites for conservation. However, the sites selected by Marxan are not necessarily those that contain high amounts of ES, but those areas that fit the three criteria mentioned above. Furthermore, while determining ES hotspots with the help of a GIS is a more or less intuitive, pragmatic and easy-to-use method, Marxan requires a substantial amount of time to prepare input data.

While we did not provide a new and standardised hotspot definition and method here, we discussed that it might be useful to recall the definition of a biodiversity hotspot and thus also consider the level of threat to ES provision in the delineation of ES hotspots. This study provides an overview of currently applied hotspot methods and should be seen as a step to trigger discussion in order to harmonise methods.

## Electronic supplementary material

Supplementary material 1 (DOCX 11 kb)
