# Supplementary material for: Spatial prioritisation for conserving ecosystem services: comparing hotspots with heuristic optimisation
Source: Landsc Ecol. 2015 Sep 2;31:431–50. doi: 10.1007/s10980-015-0258-5 (PMC4722056; doi:10.1007/s10980-015-0258-5)
Supplement: Supplementary file 1 — Supplementary material 1 (DOCX 11 kb) [file 10980_2015_258_MOESM1_ESM.docx]

Appendix 1: Parameters Marxan

Marxan input file and parameters. For abbreviations see (Game and Grantham 2008).

| General Parameters  BLM 0.005  PROP 0.5  RANDSEED -1  NUMREPS 100  Annealing Parameters  NUMITNS 1000000  STARTTEMP -1  NUMTEMP 10000  Cost Threshold  COSTTHRESH 33572  THRESHPEN1 14.0  THRESHPEN2 1.0  Program control.  RUNMODE 1  MISSLEVEL 1  ITIMPTYPE 0  HEURTYPE -1  CLUMPTYPE 0  VERBOSITY 3 |
| --- |
| Feature penalty factor  FPF: 1.0 for all features |
